# Development and Efficacy Evaluation of an Indirect ELISA Method Based on the Immunodominant Region of the Spike Protein of Porcine Epidemic Diarrhea Virus

**DOI:** 10.3390/vetsci13060524

**Published:** 2026-05-28

**Authors:** Wenyue Qiao, Huangtao He, Yutong Zhou, Biao Kong, Gerang Zeli, Shan Zhao, Qigui Yan, Yifei Lang

**Affiliations:** College of Veterinary Medicine, Sichuan Agricultural University, Chengdu 611130, China

**Keywords:** porcine epidemic diarrhea virus (PEDV), spike protein, ELISA, antibody detection

## Abstract

Porcine epidemic diarrhea virus (PEDV) is a major viral pathogen responsible for severe diarrhea and substantial economic losses in the swine industry. Characterized by its high infectivity, pathogenicity and mortality rates in neonatal piglets, PEDV has caused devastating epidemics globally over the past decade. Therefore, development of rapid, sensitive, and specific serological tests is therefore critical for effective disease surveillance and immunity evaluation. In this study, we developed and validated an indirect ELISA using the immunodominant S1^0AB^ region of the PEDV spike protein. The assay exhibited acceptable sensitivity, strong reproducibility (CV < 10%) and excellent specificity with no cross-reactivity against other common swine viruses. The test results also exhibited high concordance with virus neutralization tests (AUC = 0.913; kappa = 0.797). In addition, the ELISA was utilized to analyze 1629 field serum samples collected from southwestern China. The results indicated significant regional differences occur regarding PEDV seroprevalence and proved its potential for large-scale seroepidemiological studies. In conclusion, the S1^0AB^-based indirect ELISA developed in the present study provides a reliable, specific, and practical tool for PEDV serodiagnosis and control.

## Simple Summary

Porcine epidemic diarrhea (PED) is an acute, highly contagious enteric disease caused by porcine epidemic diarrhea virus (PEDV). PED has rapidly spread to numerous countries worldwide, causing substantial economic losses to the swine breeding industry. The initially prevalent virus strain subtype was GI, while accumulation of mutations in the viral genome has shifted the predominant virus strain subtype to the current GII, rendering commercial vaccines ineffective. To this end, timely monitoring of PEDV antibody levels is critical. In the present study, an immunodominant region of PEDV spike protein was first identified through systematic screening, while an indirect enzyme-linked immunosorbent assay (ELISA) was subsequently developed. Due to its rapidity, simplicity and sensitivity, this assay serves as an effective diagnostic tool for examination of PEDV antibodies for future vaccine immunogenicity evaluation and infection status assessment.

## 1. Introduction

Porcine epidemic diarrhea (PED) is an acute, highly contagious enteric disease caused by porcine epidemic diarrhea virus (PEDV) [1]. Clinical signs post-PEDV infection primarily include watery diarrhea and severe vomiting; mortality is exceptionally high among piglets, reaching 100% in those under seven days of age [2]. PED was first reported in the United Kingdom in 1977 [3] and subsequently spread rapidly worldwide, affecting as many as 38 U.S. states [4]. Continuous outbreaks also occurred in Japan, Canada, Mexico, and Colombia, resulting in substantial economic losses to the global swine industry and posing serious challenges for disease control and prevention [5].

As a member of the *Alphacoronavirus* genus [6], PEDV is an enveloped virus with a positive-sense RNA genome approximately 28 kb in length [7]. The PEDV genome encodes several nonstructural proteins and four main structural proteins, namely the spike (S), envelope (E), membrane (M), and nucleocapsid (N) [8]. The S protein is the main structural viral protein that mediates both viral attachment and the subsequent membrane fusion [9]. Moreover, as the primary target of neutralizing antibodies, the S protein is often used for the determination of evolutionary relationships among PEDV isolates and development of diagnostic assays and vaccine strategies [10,11]. The S protein consists of two subunits, namely the N-terminal S1 subunit that mediates viral attachment to host cell receptors and the C-terminal S2 subunit that arbitrates fusion between the viral and host cell membranes [12]. The S1 subunit can be further divided into five domains (S1^0^, S1^A^-S1^D^) [13]. Among these, the N-terminal S1^0^ domain is unique to alphacoronaviruses [14], exhibits sialic acid-binding and neutralizing activities, and serves as a key determinant of PEDV pathogenicity [15,16]. The S1^B^ domain primarily mediates binding to host cell receptors and possesses neutralizing activity, making it an important target for vaccine development [17,18].

Multiple serological assays have been developed for detecting PEDV antibodies, including immunofluorescence assays (IFAs), enzyme-linked immunosorbent assays (ELISAs), colloidal gold immunochromatography assays (GICAs), and virus neutralization tests (VNTs) [19]. Based on the measurement of antibody titers assessed by the reduction in cytopathic effects or plaque formation on susceptible cell lines, VNTs are generally regarded as the “golden standard”. However, their implementation requires the culture of infectious PEDV samples in a biosafety laboratory, along with tissue culture expertise and extended incubation periods, henceforth limiting their usage for large-scale surveillance. Therefore, developing safe, accessible assays that yield results consistent with those of VNTs is essential for PEDV serology. Compared with other serological assays, ELISA offers higher specificity and sensitivity, is operationally simple and rapid, and imposes minimal technical requirements on personnel, making it highly suitable for large-scale clinical sample testing for PEDV. ELISA is a technique primarily based on specific antigen—antibody binding reactions and high-sensitivity chromogenic reactions catalyzed by enzymes; common methods include indirect, sandwich, and competitive assays [20]. Given the continuous emergence of novel PEDV variants, particularly the S-INDEL strains that have been increasingly identified in China, development of ELISA methods capable of detecting antibodies against diverse genotypes is of particular importance. This method can be used for monitoring PEDV antibody levels in serum and also for evaluating vaccine-induced immune responses or directly detecting PEDV antigens in clinical specimens such as intestinal contents or feces from diseased pigs [21,22].

In the present study, we expressed chimeric PEDV S1^B^ and S1^0AB^ proteins using a HEK-293T eukaryotic expression system and individually employed them as coating antigens to identify the optimal detection antigen derived from the PEDV spike protein. Subsequently, we developed an indirect ELISA based on this optimal detection antigen, which demonstrated high concordance and agreement with the results of the virus neutralization test (VNT). The established indirect ELISA was next used to detect PEDV antibodies in 1629 clinical porcine serum samples collected from southwestern regions of China. Generally, our indirect ELISA approach is suitable for the epidemiological surveillance of PEDV in clinical settings and provides a rapid and effective diagnostic tool for the prevention and control of this disease.

## 2. Materials and Methods

### 2.1. Cells, Viruses, Serum, and Clinical Samples

HEK-293T and Vero cells preserved by the College of Veterinary Medicine, Sichuan Agricultural University, were cultured in Dulbecco’s modified Eagle’s medium (DMEM; Gibco, Grand Island, NY, USA) supplemented with 10% (*w*/*v*) fetal bovine serum (FBS) (ExCell Bio, Suzhou, China), penicillin (100 IU/mL) and streptomycin (100 μg/mL). The cells were cultured at 37 °C, 5% CO_2_. The PEDV/SC/2020 strain was isolated and preserved by the College of Veterinary Medicine, Sichuan Agricultural University [23].

PEDV-negative serum was obtained from specific-pathogen-free (SPF) pigs. PEDV-positive serum was collected from pigs infected on commercial farms and subsequently confirmed in the laboratory using a commercially available diagnostic kit.

A total of 1629 clinical serum samples were collected in 2026 from swine farms in southwestern China (Sichuan, Chongqing, Yunnan, and Guizhou Provinces) and stored at −20 °C in the laboratory.

### 2.2. Recombinant Plasmid Construction

Recombinant plasmids expressing chimeric PEDV S1^B^ and S1^0AB^ proteins were constructed as described previously [23]. In brief, C-terminal strep tags were added to the coding sequences of the PEDV S1^B^ (aa505-639) and S1^0AB^ (aa1-639) domains to facilitate subsequent protein purification, which were subsequently cloned and inserted into the pcDNA-3.1 expression plasmid. The constructed plasmid was verified by sequencing.

### 2.3. Recombinant Protein Expression and Purification

S1^B^-strep and S1^0AB^-strep expression plasmids conjugated to PEI (Polysciences, Warrington, PA, USA) were transfected into HEK-293T cells for the production of recombinant proteins. At 5 days post-transfection, the cell supernatant containing the recombinant protein was collected and centrifuged, after which the proteins were purified with Strep-tag^®^II (IBA Lifesciences, Inc., Göttingen, Germany) according to the manufacturer’s instructions. Purified proteins were quantified by NanoDrop spectrophotometry (Thermo Fisher Scientific, Inc., Waltham, MA, USA) followed by SDS-PAGE and Western blot validation. The purified proteins were stored at −80 °C prior to antigen coating.

### 2.4. Western Blot Analysis

For Western blot analysis, 2 µg of protein was blotted onto a 0.45 µm polyvinylidene fluoride (PVDF) membrane (Absin Bioscience Inc., Shanghai, China). The membranes were incubated in 5% blocking buffer (0.05% PBST (20 × PBS, Sangon Biotech Co., Ltd., Shanghai, China; Tween-20, Xilong Scientific, Guangzhou, China) containing 5% skim milk powder (Protifar, Nutricia, Zoetermeer, The Netherlands) at 37 °C for 2 h. After blocking, the membranes were incubated with primary antibodies (anti-Strep-Tag II monoclonal antibody, 1:2000; Abbkine Scientific Co., Ltd., Atlanta, GA, USA), followed by horseradish peroxidase (HRP)-conjugated rabbit anti-mouse IgG (Sangon Biotech Co., Ltd., China; 1:1000). Finally, the membranes were examined using SuperSignal West Pico PLUS (Thermo Fisher Scientific Inc., Waltham, MA, USA) according to the manufacturer’s instructions.

### 2.5. Virus Neutralization Test (VNT)

The viral stock was first diluted in maintenance medium (DMEM containing 5 μg/mL trypsin) to yield PEDV suspensions at final concentrations of 2000 TCID_50_/mL, 200 TCID_50_/mL, 20 TCID_50_/mL, and 2 TCID_50_/mL. Serum samples were serially diluted twofold in the same maintenance medium starting from a 1:8 dilution in 96-well plates. Afterward, an appropriate volume of the PEDV suspension was added to each well to achieve a final virus concentration of 100 TCID_50_/mL, followed by incubation at 37 °C for 1 h. The serum–virus mixtures were subsequently transferred onto Vero cells cultured in 96-well plates and incubated at 37 °C for 2 h. After incubation, the supernatant was removed, and the cell monolayers were washed three times with sterile phosphate-buffered saline (PBS). Finally, 200 μL of maintenance medium was added to each well. Cytopathic effects (CPEs) were monitored daily, and the serum neutralizing titer was calculated. Each serum sample was analyzed independently in triplicate.

### 2.6. PEDV S Protein Dominant Detection Antigen Screening

The PEDV S1^B^ and S1^0AB^ recombinant proteins were each used as ELISA coating antigens. The OD_450nm_ values of 100 pig serum samples were then measured and compared with the results of virus neutralization tests. The receiver operating characteristic (ROC) curve was plotted using SPSS 24.0 software, and the area under the curve (AUC) was calculated. The detailed ELISA procedure is as follows:

(1) Antigen coating: The antigen coating concentration was 1 μg/mL, and each recombinant protein was diluted in PBS and coated at 4 °C for 16 h.

(2) Blocking: The 96-well microplate was washed four times with 0.05% PBST and then blocked with 5% skim milk at 37 °C for 2 h.

(3) Primary antibody: After blocking, the 96-well microplates were washed four times with 0.05% PBST, and 100 μL of 1:100 diluted serum primary antibody was added to each well and incubated at 37 °C for 1 h.

(4) Secondary antibody: After primary antibody incubation, the 96-well microplate was washed four times with 0.05% PBST, and 100 μL of 1:4000 diluted enzyme-labeled secondary antibody was added to each well and incubated at 37 °C for 1 h.

(5) Color development: After secondary antibody incubation, the 96-well microplate was washed four times with 0.05% PBST, and 100 μL of TMB substrate was added to each well and incubated at room temperature in the dark for 10 min. Finally, 100 μL of 12.5% H_2_SO_4_ was added to stop the reaction, and the OD_450nm_ value was read using a microplate reader.

### 2.7. Establishment of an Indirect ELISA Method and Optimization of Conditions

The ELISA conditions were systematically optimized in 96-well plates coated with high-binding polystyrene, and the protein coating concentration (0.5–2 μg/mL), serum dilution ratio (1:100–1:3200), and coating duration (16 h at 4 °C) were tested. Further optimization included determining the optimal serum incubation time (30–120 min), the best secondary antibody dilution (1:1000, 1:2000, 1:4000, and 1:8000), the optimal secondary antibody incubation time (30–120 min), and the TMB substrate incubation time (5–20 min) to identify the best detection conditions. Under the optimized conditions, the P/N values of each group were compared, and the highest P/N value was selected as the corresponding optimal condition.

### 2.8. Determination of the Cutoff Value of the ELISA

To determine the cutoff values for positive and negative results in this indirect ELISA, a total of 50 neutralization-corrected negative porcine serum samples were selected, and their OD_450nm_ values were measured using the optimized indirect ELISA. The mean (X) and standard deviation (SD) were calculated. Serum samples with OD_450nm_ < X + 2 SD were classified as negative, those with OD_450nm_ > X + 3 SD were classified as positive, and those falling between these two values were classified as suspicious.

### 2.9. Sensitivity and Reproducibility of the ELISA

To evaluate the sensitivity of this method, ten negative serum samples and twenty-five positive serum samples were selected and serially diluted in a twofold series from 1:800 to 1:102,400. The OD_450nm_ values were measured using the established indirect ELISA method. To subsequently assess the reproducibility of this method, two sets of tests were conducted: within-batch and between-batch replicates. Finally, the coefficient of variation was calculated to determine the reproducibility of this method.

### 2.10. Analysis of the Specificity and Agreement Rate of the ELISA

The specificity of the indirect ELISA method established in the aforementioned experiments was evaluated by simultaneously testing serum samples positive for PEDV, PCV2, PCV3, CSFV, PRV and PRRSV. The OD_450nm_ values of 100 clinical porcine serum samples whose VNTs were corrected were subsequently measured, and ROC curves were plotted and kappa values calculated.

### 2.11. Validation of the ELISA Using Clinical Samples

To evaluate the performance of this indirect ELISA method for testing clinical samples, a total of 1629 serum samples were collected from pig farms in parts of southwestern China for testing.

## 3. Results

### 3.1. Characterization of Recombinant Proteins

**Figure 1:**
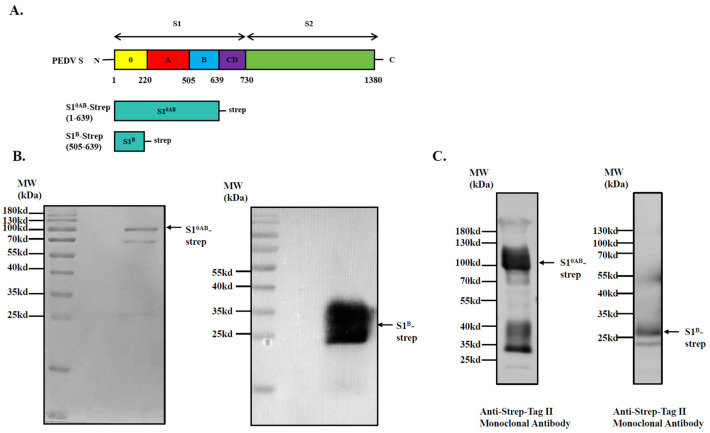
Preparation and characterization of recombinant protein. (**A**) Schematic diagram of the PEDV S protein genome. The five domains of S1 and their respective boundaries: 0 (yellow) (aa1–220), A (red) (aa220–505), B (blue) (aa505–639) and CD (purple) (aa639–730). Below are schematic diagrams of the sequences of the recombinant proteins S1^0AB^-strep and S1^B^-strep used in this study; (**B**) S1^0AB^-strep and S1^B^-strep recombinant proteins were detected by SDS-PAGE; (**C**) S1^0AB^-strep and S1^B^-strep recombinant proteins were detected by Western blotting. The numbers on the left are molecular masses (in kilodaltons). (See Figure S1).

To identify the immunodominant region within the PEDV S protein, the S1^0AB^ and S1^B^ proteins of PEDV were first expressed, purified and identified using SDS-PAGE and Western blot analysis. All S1^0AB^ and S1^B^ proteins displayed electrophoretic mobilities corresponding to their expected molecular weights. The recombinant proteins were verified by SDS-PAGE and Western blotting, which revealed that they migrated to the expected molecular weights and bound specifically to anti-streptavidin antibodies, indicating that the recombinant S1^0AB^ and S1^B^ proteins were successfully expressed and purified (Figure 1B,C).

### 3.2. PEDV S Protein Dominant Detection Antigen Screening

**Figure 2:**
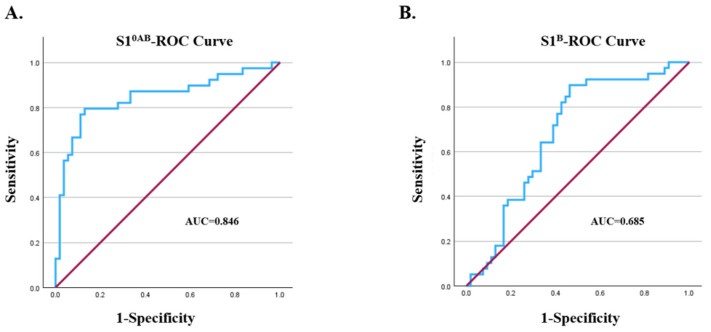
The ROC curve of the correlation between ELISA and VNT was plotted using SPSS software, and the area under the curve (AUC) was calculated. (**A**) ROC curve of S1^0AB^ indirect ELISA, AUC = 0.846; (**B**) ROC curve of S1^B^ indirect ELISA, AUC = 0.685.

In order to investigate whether the S1^B^ or S1^0AB^ domain is the dominant antigen of the PEDV S protein, this study compared the results of two indirect ELISAs with the results of serum neutralization tests. ROC curves were plotted, and AUC values were calculated using SPSS software. The results revealed that the AUC for the S1^0AB^ indirect ELISA was 0.846, with a 95% confidence interval of [0.757, 0.934]. The AUC of the S1^B^ indirect ELISA was 0.685, with a 95% confidence interval of [0.575, 0.794] (Figure 2). These findings demonstrate that S1^0AB^ is a more suitable antigen for the detection of the PEDV S protein.

### 3.3. Establishment of an Indirect ELISA Method and Optimization of Conditions

**Figure 3:**
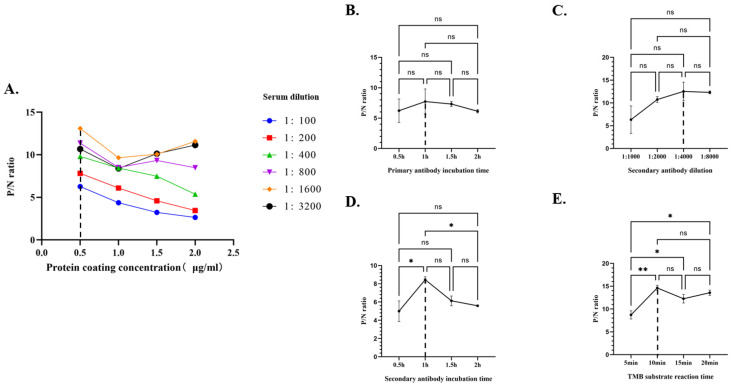
Optimization of indirect ELISA detection method conditions. (**A**) Checkerboard titration method to determine antigen coating concentration and serum dilution, with results presented as P/N values; (**B**) optimization of serum incubation time conditions (*p* > 0.05), presented using P/N values; (**C**) optimization of secondary antibody dilution conditions (*p* > 0.05), presented using P/N values; (**D**) optimization of secondary antibody incubation time conditions (*p* < 0.05), presented using P/N values; (**E**) optimization of the TMB substrate incubation time conditions (*p* < 0.01), presented using P/N values. The dashed lines in the figure indicate the optimal conditions.*, *p* < 0.05; **, *p* < 0.01; ns, not significant.

This study first optimized the conditions for indirect ELISA detection. The optimized ELISA conditions were as follows: the optimal antigen concentration was 0.5 μg/mL (Figure 3A), the optimal serum dilution was 1:1600 (Figure 3A), the optimal serum incubation time was 1 h (Figure 3B), the optimal dilution of the labeled secondary antibody was 1:4000 (Figure 3C), the optimal incubation time for the labeled secondary antibody was 1 h (Figure 3D), and the optimal reaction time for the TMB substrate was 10 min (Figure 3E).

Secondly, in order to investigate the cutoff value of this indirect ELISA method, in this study, 50 serum samples negative for PEDV neutralizing antibodies were tested using ELISA; the results are shown in Table 1. The mean OD_450nm_ value for the 50 PEDV neutralizing antibody-negative serum samples was X = 0.167, with a standard deviation (SD) of 0.042; X + 2 SD = 0.251; and X + 3 SD = 0.293. Consequently, when the OD_450nm_ value of a test sample is <0.251, it is classified as negative; when the OD_450nm_ value of a test sample is >0.293, it is classified as positive.

### 3.4. Sensitivity and Reproducibility of the ELISA

**Figure 4:**
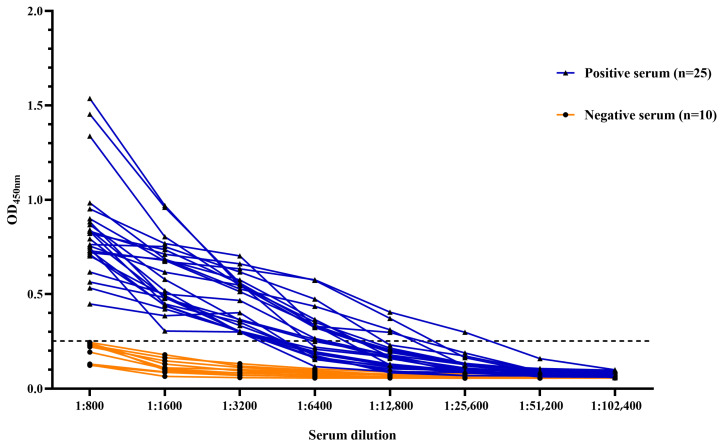
The dilution range of the PEDV serum was between 1:800 and 1:102,400, and the sensitivity of this method was analyzed. The dashed line in the figure represents the positive threshold.

On the basis of the optimized ELISA conditions described above, we evaluated the sensitivity and stability of the method. In the sensitivity test (Figure 4), when serum was diluted to 1:3200, the OD_450nm_ value of the positive serum remained above the positive threshold, and the minimum detection limit was up to 1:25,600, indicating that the method has good sensitivity. In the stability tests, the coefficient of variation (CV) for the intra-assay replicates ranged from 0.93% to 3.2%, all of which were less than 5% (Table 2), whereas the CV for the inter-assay replicates ranged from 0.79% to 9.85%, all of which were less than 10% (Table 3), indicating that this indirect ELISA method is stable.

### 3.5. Analysis of the Specificity and Agreement Rate of the ELISA

**Figure 5:**
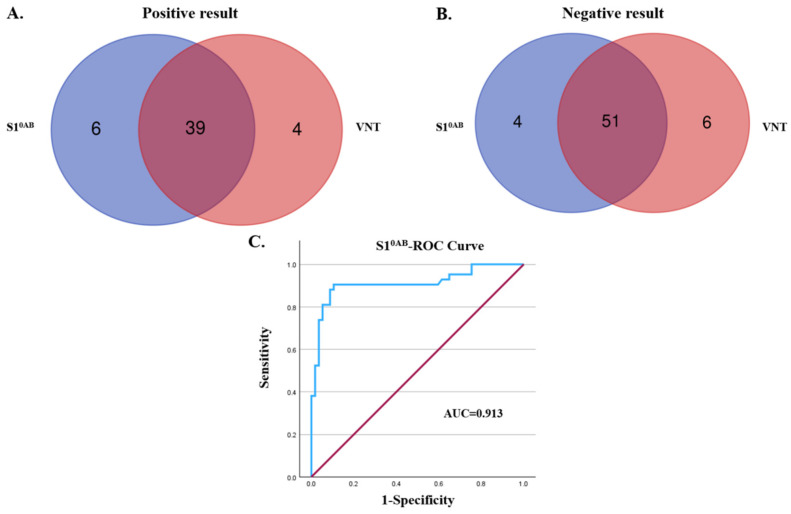
Venn diagrams and ROC curve analysis of ELISA and VNT results in pig serum samples. (**A**) The overlap in the number of positive samples between the ELISA and VNT is illustrated using a Venn diagram; (**B**) the overlap in the number of negative samples between the ELISA and VNT tests is illustrated using a Venn diagram; (**C**) ROC curve analysis of the correlation between the ELISA and VNT test results; AUC = 0.913.

This study uses the established indirect ELISA method to detect positive sera of different pig pathogens, in order to analyze the specificity of this method. Specificity analysis revealed that this method did not cross-react with other common porcine viral pathogens (Table 4). This indicates that the indirect ELISA method established in this study has suitable specificity. In order to analyze the feasibility of this method, VNT was used as the gold standard, where a total of 100 clinical pig serum samples were tested and 43 and 57 samples were determined to be positive and negative for neutralizing antibodies. These VNT results serve as a reference for evaluating the feasibility of the ELISA method. Concurrently, the OD_450nm_ values of these 100 samples were measured using the PEDV indirect ELISA method established in this study. Details of the positive and negative samples are shown in Table 5 and presented as Venn diagrams (Figure 5A,B). The ROC curves were subsequently plotted, and the kappa values were calculated using SPSS software to analyze the test results. The results revealed an area under the curve (AUC) of 0.913, with the 95% confidence interval of [0.848, 0.978] (Figure 5C) and a concordance rate (kappa) of 0.797 (*p* < 0.001, Z = 7.978). In summary, the established indirect ELISA method demonstrated good sensitivity and specificity and exhibited a high degree of agreement with the neutralization assay.

### 3.6. Clinical Sample Detection by ELISA

**Figure 6:**
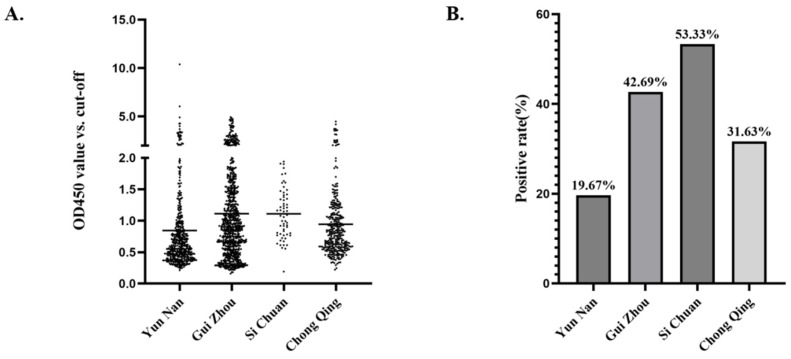
Epidemiology of PEDV serology in pigs in southwest China (2026). (**A**) Distribution map of 1629 pig serum samples; (**B**) bar chart showing the positive rate of PEDV antibodies in pig serum from different regions.

In 2026, 1629 serum samples were collected from pig herds at selected pig farms in four provinces and tested using the method established in this study. The results indicated an overall seroprevalence of 34.07%. However, regional analysis revealed variations in seroprevalence across different regions. Among those, the region with the highest seroprevalence was Sichuan (53.33%), followed by Guizhou (42.69%), whereas the remaining regions exhibited lower seroprevalence, namely, Chongqing (31.63%) and Yunnan (19.67%) (Figure 6).

## 4. Discussion

Prior to 2010, PEDV was effectively controlled in China through the use of an oil-emulsified inactivated vaccine developed on the basis of the CV777 strain, with the virus circulation only occur in certain regions. However, after 2010, the continuous accumulation of insertions and mutations in the S gene of PEDV resulted in greater pathogenicity among variant strains, which became the primary pathogen of PED [24] and ultimately led to widespread outbreaks of PEDV infection on Chinese pig farms in 2011. Since then, PEDV has become one of the most common viral diarrhea pathogens in the current swine farming industry. Although numerous commercial vaccines are currently in use, none can provide effective and complete cross-protection. Therefore, establishing methods for detecting PEDV antibody levels and monitoring these levels in pig herds in a timely and effective manner is crucial for PEDV prevention and control.

Currently, common serological diagnostic methods for PEDV primarily include IFA, VNT and ELISA. Both IFA and VNT require cell culture in specific environments and consequently impose high demands on testing equipment, environmental conditions and operator skills; therefore, they are unsuitable for rapid, large-scale testing in clinical settings. In contrast, the ELISA method offers advantages such as rapid reaction times, ease of operation, high sensitivity, good specificity, minimal equipment requirements, and the ability to conduct large-scale antibody testing on clinical serum samples. Consequently, it is frequently employed in clinical diagnostics.

Antigen selection is the key aspect of the ELISA process. With the advantage of high detection sensitivity and simple preparation, whole-virus antigens are often selected as coating antigens. However, their specificity may be compromised because of existing cross-reactivity with antibodies from other closely related viruses. In contrast, detection methods using viral proteins could avoid these drawbacks. Currently, the PEDV N protein is frequently used in commercial ELISA kits due to its high expression level and strong conservation among different PEDV strains [25]. However, it is also shown that the PEDV N protein exhibits cross-reactivity with the N proteins of other coronaviruses, such as TGEV and PDCoV [26,27]. In contrast, the S protein, especially the S1^0AB^ region used in the present study, contains major neutralizing epitopes and elicits longer-lasting antibodies post virus infection, making it more suitable for PEDV antibody detection [28]. Gerber et al. developed an S1-based indirect ELISA to detect anti-PEDV IgG and IgA antibody levels in sow serum and colostrum for the immune status assessment, achieving 100% sensitivity for serum and 94% for colostrum, with no cross-reactivity to other porcine coronaviruses [29].

Among the S1^0AB^ region used in this study, the S1^0^ domain possesses both sialic acid-binding activity and neutralizing activity and is a key determinant of PEDV pathogenicity. The S1^A^ domain has also been shown to contain linear epitopes and to play a role in stabilizing the S1^0^ domain. The S1^B^ domain, on the other hand, was initially identified as the receptor-binding domain (RBD) and serves as the primary target for neutralizing antibodies [17]. Eukaryotic expression systems in mammalian cells can ensure precise protein folding and appropriate posttranslational modifications; the proteins expressed thereby also exhibit neutralizing activity, which is highly important for the development of serological assays [30]. The Strep tag enables efficient protein purification with minimal impact on protein structure and activity, and its mild purification conditions maximize the retention of protein bioactivity, thereby yielding high-purity target proteins [31]. Consequently, in the present study, we designed expression plasmids for PEDV S1^B^ and S1^0AB^ fused with the Strep tag and expressed them using the HEK-293T eukaryotic expression system. We compared these two recombinant proteins and subsequently optimized an indirect ELISA method on the basis of the identified dominant antigens. Finally, the established ELISA method was utilized to test a large number of clinical samples, providing preliminary data on PEDV antibody levels in certain regions of southwestern China.

The results show that the PEDV S1^0AB^ region is the suitable antigen for PEDV antibody detection, while the hereby established indirect ELISA method demonstrated good sensitivity, with positive sera remaining positive at a dilution of 1:3200. Although in repetitive experiments, the CV value of the eighth group in the inter-batch reproducibility test was relatively high, close to 10%, the range was still controlled within 10%, and the CV values of other groups remained at a low level, meeting the common standards for preliminary method acceptability in most methodological validation guidelines. Therefore, the method still has effective reproducibility. Furthermore, this indirect ELISA method showed no cross-reactivity with other common porcine pathogens. Furthermore, this method demonstrated a high degree of agreement (kappa = 0.797) and consistency (AUC = 0.913) with the neutralization test. Finally, we used the established indirect ELISA method to test serum samples from selected pig farms in Yunnan, Guizhou, Sichuan and Chongqing. The results revealed that the anti-PEDV antibody positivity rate was highest in Sichuan (53.33%) and lowest in Yunnan (19.67%), with Guizhou (42.69%) and Chongqing (31.63%) in the middle, demonstrating a decreasing trend from Sichuan to the surrounding provinces. The differences in PEDV seroprevalence rates among these four regions may be mainly related to factors such as breeding density, farming patterns, transportation, and geographic and climatic conditions. Among them, Sichuan has the highest PEDV antibody positive rate, likely due to the high breeding density, a humid climate that favors virus survival, and the frequent pig transport risking PEDV transmission. Neighboring Guizhou and Chongqing showed intermediate rates, likely influenced by Sichuan. Yunnan had the lowest rate, attributed to its mountainous terrain, scattered farming, and dry–hot plateau environment unfavorable for PEDV. Moreover, differences in immunization practices in different regions and inconsistencies in farm types sampled may also affect the positive rates across the four regions. Further tests such as RT-PCR and genomic sequencing shall be conducted to identify the predominant circulating PEDV strains in those regions, thereby enabling targeted prevention and control measures.

This study first screened the dominant antigen of the PEDV S protein. Based on this antigen, an indirect ELISA detection method was established and the sensitivity, specificity, and feasibility of this method were investigated. The comprehensive results showed that this method has good sensitivity and specificity and also shows good consistency with the neutralization test. In the detection of a large number of clinical samples, the positive rates of PEDV antibodies in different regions varied to some extent, with Sichuan Province having the highest PEDV antibody-positive rate. Meanwhile, this study also has its limitations. Firstly, despite using the immunodominant S1^0AB^ as a coating antigen, the developed assay cannot completely rule out the possible cross-reactivity with antibodies against other swine alphacoronaviruses, such as TGEV or SADS-CoV. In addition, the stability and reproducibility of the ELISA method under different storage and operational conditions demand additional investigation. Future work will focus on the validation of this method with a broader panel of clinical samples under different laboratory settings, henceforth improving its ability to detect antibodies against emerging PEDV variants. Lastly, current PEDV vaccines used in the swine industry include inactivated and attenuated vaccines, both containing complete viral structural proteins. Given the fact that antibodies targeting the S protein persist longer in vaccinated or infected pigs, the ELISA method developed in this study cannot be used for differential diagnosis. Taken together, despite those limitations, the newly developed indirect ELISA detection method has the potential for the rapid detection of PEDV antibodies in a large number of clinical samples.

## 5. Conclusions

In summary, the developed indirect ELISA method provides a suitable platform for antibody screening in clinical samples. It can be implemented to determine the levels of PEDV antibodies in pig sera and monitor PEDV prevalence. In addition, it does not require complex experimental conditions or procedures, thereby providing an effective experimental method for PEDV monitoring and control. This study also provides important data support for differentiated prevention and control as well as precise management of PEDV in southwest China.

## Figures and Tables

**Table 1:** Determination of the cutoff value.

Number	1–10	11–20	21–30	31–40	41–50
OD_450nm_	0.116	0.125	0.143	0.181	0.215
	0.116	0.126	0.145	0.182	0.216
	0.117	0.127	0.145	0.187	0.22
	0.118	0.128	0.149	0.191	0.23
	0.118	0.133	0.156	0.193	0.235
	0.122	0.135	0.158	0.194	0.237
	0.123	0.135	0.162	0.196	0.238
	0.124	0.136	0.167	0.2	0.243
	0.124	0.14	0.169	0.201	0.244
	0.125	0.14	0.172	0.203	0.246

**Table 2:** Results of the intro-batch duplicability test.

Number	OD_450nm_ Value of Each Replicate Well			Average	SD	CV (%)
	1	2	3			
1	0.078	0.075	0.078	0.077	0.00198	2.58
2	0.093	0.097	0.092	0.094	0.00269	2.86
3	0.123	0.116	0.122	0.121	0.00386	3.20
4	0.075	0.074	0.075	0.075	0.00070	0.93
5	1.388	1.352	1.381	1.374	0.19088	1.39
6	0.550	0.522	0.550	0.541	0.01617	2.99
7	1.181	1.159	1.110	1.150	0.03635	3.16
8	0.536	0.556	0.558	0.550	0.01217	2.21

**Table 3:** Results of the inter-batch duplicability test.

Number	OD_450nm_ Value of Each Replicate Well			Average	SD	CV (%)
	1	2	3			
1	0.077	0.074	0.082	0.078	0.00403	5.18
2	0.094	0.093	0.093	0.093	0.00073	0.79
3	0.121	0.131	0.127	0.126	0.00540	4.28
4	0.075	0.072	0.066	0.071	0.00446	6.28
5	1.374	1.401	1.556	1.444	0.09853	6.82
6	0.541	0.520	0.594	0.552	0.03832	6.95
7	1.150	1.213	1.340	1.234	0.09699	7.86
8	0.550	0.578	0.663	0.597	0.05882	9.85

**Table 4:** Results of the specificity test.

Serum	OD_450nm_	Result
PCV2+	0.238	-
PCV3+	0.181	-
CSFV+	0.126	-
PRV+	0.14	-
PRRSV+	0.125	-
PEDV+	0.45	+
PEDV−	0.187	-

**Table 5:** Detection of anti-PEDV antibodies in pig serum samples.

S1^0AB^-ELISA	VNT		Total
	Positive Sample No.	Negative Sample No.	
Positive sample no.	39	6	45
Negative sample no.	4	51	55
Total	43	57	100

## Data Availability

The original contributions presented in this study are included in the article/Supplementary Material. Further inquiries can be directed to the corresponding author.

## Supplementary Materials

The following supporting information can be downloaded at: https://www.mdpi.com/article/10.3390/vetsci13060524/s1, Figure S1: Original figures.
