# Supplementary figures and images for: Development and Efficacy Evaluation of an Indirect ELISA Method Based on the Immunodominant Region of the Spike Protein of Porcine Epidemic Diarrhea Virus
Source: Vet Sci. 2026 May 28;13(6):524. doi: 10.3390/vetsci13060524 (PMC13308466; doi:10.3390/vetsci13060524)

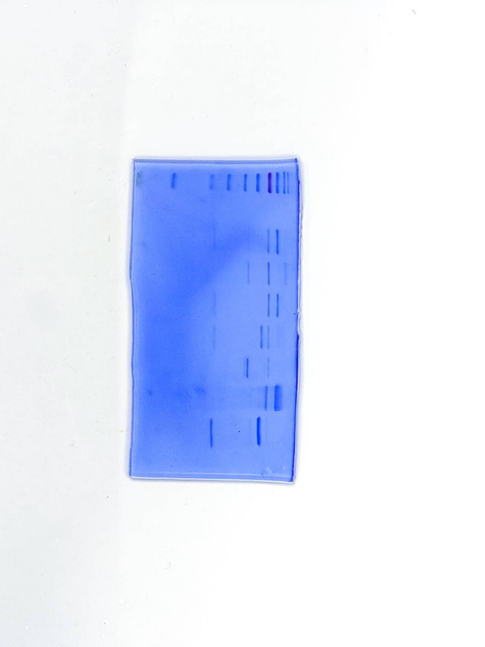

Supplement: Supplementary file 1 [file vetsci-13-00524-s001.zip › Figure S1/Figure S1.B-1.png]

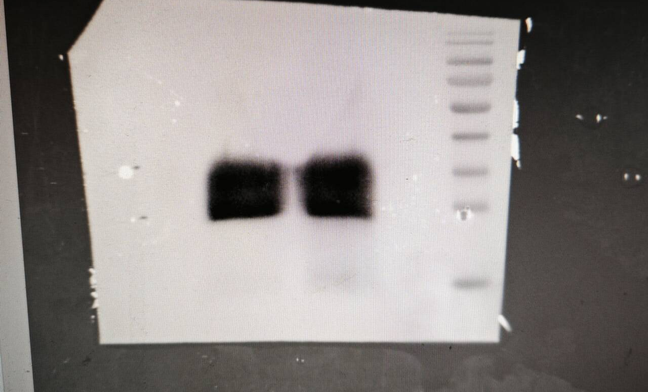

Supplement: Supplementary file 1 [file vetsci-13-00524-s001.zip › Figure S1/Figure S1.B-2.png]

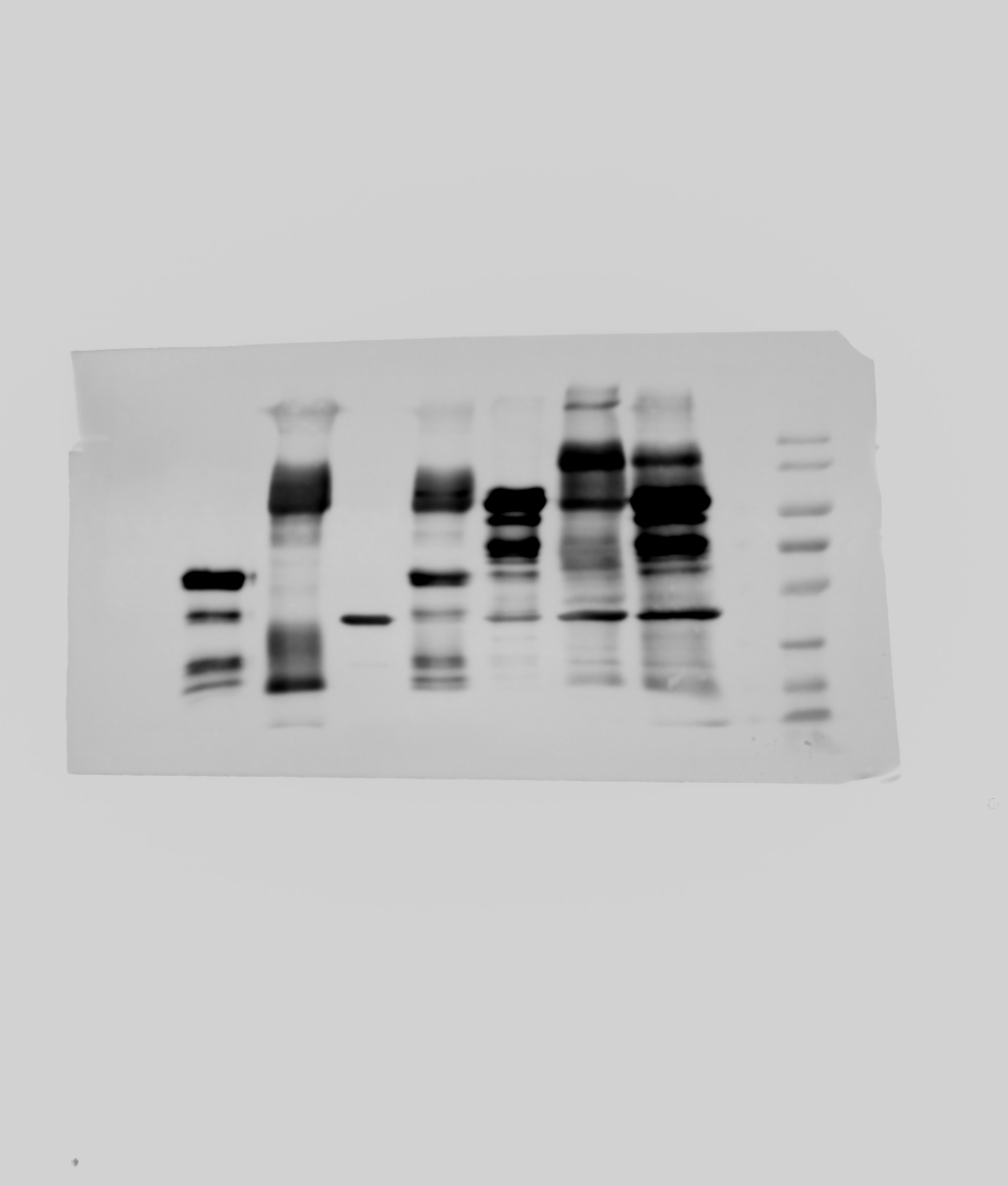

Supplement: Supplementary file 1 [file vetsci-13-00524-s001.zip › Figure S1/Figure S1.C-1.png]

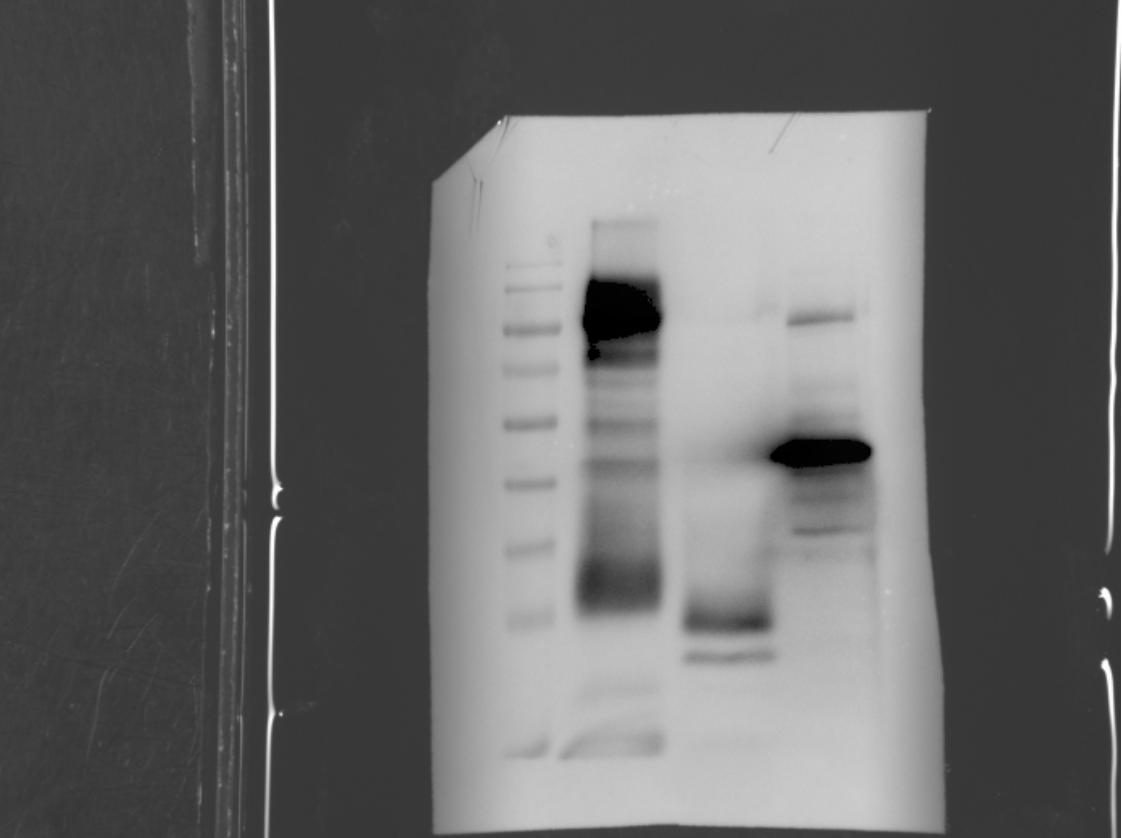

Supplement: Supplementary file 1 [file vetsci-13-00524-s001.zip › Figure S1/Figure S1.C-2.png]
